# Cost utility analysis of intramedullary nailing and skeletal traction treatment for patients with femoral shaft fractures in Malawi

**DOI:** 10.1080/17453674.2021.1897927

**Published:** 2021-03-24

**Authors:** Linda Chokotho, Claire A Donnelley, Sven Young, Brian C Lau, Hao-Hua Wu, Nyengo Mkandawire, Jan-Erik Gjertsen, Geir Hallan, Kiran J Agarwal-Harding, David Shearer

**Affiliations:** a Department of Surgery, College of Medicine, University of Malawi ;; b Department of Clinical Medicine, University of Bergen , Bergen , Norway ;; c Institute for Global Orthopedics and Traumatology, Orthopedic Trauma Institute, University of California San Francisco , San Francisco , CA , USA ;; d Department of Orthopedic Surgery, Haukeland University Hospital , Bergen , Norway ;; e Department of Surgery, Kamuzu Central Hospital , Lilongwe , Malawi ;; f Department of Orthopedic Surgery, Duke University Medical Centre , Durham , NC , USA ;; g School of Medicine, Flinders University , Adelaide , Australia ;; h Harvard Global Orthopaedics Collaborative, Harvard Combined Orthopaedic Residency Program, Massachusetts General Hospital , Boston , MA , USA

## Abstract

Background and purpose — In Malawi, both skeletal traction (ST) and intramedullary nailing (IMN) are used in the treatment of femoral shaft fractures, ST being the mainstay treatment. Previous studies have found that IMN has improved outcomes and is less expensive than ST. However, no cost-effectiveness analyses have yet compared IMN and ST in Malawi. We report the results of a cost-utility analysis (CUA) comparing treatment using either IMN or ST.

Patients and methods — This was an economic evaluation study, where a CUA was done using a decision-tree model from the government healthcare payer and societal perspectives with an 1-year time horizon. We obtained EQ-5D-3L utility scores and probabilities from a prospective observational study assessing quality of life and function in 187 adult patients with femoral shaft fractures treated with either IMN or ST. The patients were followed up at 6 weeks, and 3, 6, and 12 months post-injury. Quality adjusted life years (QALYs) were calculated from utility scores using the area under the curve method. Direct treatment costs were obtained from a prospective micro costing study. Indirect costs included patient lost productivity, patient transportation, meals, and childcare costs associated with hospital stay and follow-up visits. Multiple sensitivity analyses assessed model uncertainty.

Results — Total treatment costs were higher for ST ($1,349) compared with IMN ($1,122). QALYs were lower for ST than IMN, 0.71 (95% confidence interval [CI] 0.66–0.76) and 0.77 (CI 0.71–0.82) respectively. Based on lower cost and higher utility, IMN was the dominant strategy. IMN remained dominant in 94% of simulations. IMN would be less cost-effective than ST at a total procedure cost exceeding $880 from the payer’s perspective, or $1,035 from the societal perspective.

Interpretation — IMN was cost saving and more effective than ST in the treatment of adult femoral shaft fractures in Malawi, and may be an efficient use of limited healthcare resources.

The incidence of femoral shaft fractures in low- and middle-income countries (LMICs) is estimated to range from 16 to 46 per 100,000 people per year (Agarwal-Harding et al. 2015). In Malawi, which has 17.5 million inhabitants (National Statistical Office of Malawi 2019), a recent study estimated the prevalence of femoral shaft fractures at 1.4 per 100,000 people and incidence of 27 per 100,000 people per year (Agarwal-Harding et al. 2020), translating into approximately 4,700 fractures annually. For comparison, the annual incidence of femoral shaft fractures in Sweden is one third of that in Malawi (personal communication, Michael Möller, The Swedish Fracture Registry). The goal of treatment for these fractures is to achieve stability at the fracture site, thereby promoting union and painless weight-bearing, and allowing early patient rehabilitation. Treatment with intramedullary nailing (IMN) achieves this goal earlier and more consistently than skeletal traction (ST), and has become the gold standard for managing these fractures in high-income countries. In Malawi, however, treatment using ST, requiring patient immobilization in bed for at least 6 weeks, remains the mainstay treatment.

Femoral shaft fractures do not only affect physical function, but also the patient’s social and psychological well-being (Haug et al. 2017, Kohler et al. 2017). Accordingly, better treatment of these fractures should improve quality of life by improving not only physical function but also social and psychological functions. A quality adjusted life year (QALY) is an appropriate measure of outcome as it includes both quantity and quality of life (Stothers 2006). Studies from Malawi and elsewhere have found that treatment with IMN is less costly compared with ST (Gosselin et al. 2009, Opondo et al. 2013, Kamau et al. 2014, Diab et al. 2019). However, these studies did not assess the effectiveness of these 2 treatment modalities using a generic outcome measure such as the QALY. As such it remains unclear which modality represents a better use of limited healthcare resources in terms of costs and QALYs gained. Malawi is a low-income country in Southern Africa with a gross domestic product (GDP) per capita of only US$380 (World Bank 2019a). In a resource-limited setting like Malawi, appropriate resource allocation to ensure optimization of the healthcare budget is a priority. Cost-effectiveness analyses of health care interventions can provide the necessary evidence needed to change clinical practice, funding, and policies for the better.

We evaluated the cost-effectiveness of IMN versus ST in the treatment of femoral shaft fractures in Malawi using QALYs as a measure of effectiveness, to determine which treatment modality best represents efficient use of healthcare resources from government healthcare payer and societal perspectives.

## Patients and methods

### Design and setting

This study is a cost-utility analysis (CUA) comparing IMN with ST for treatment of adult femoral shaft fractures in Malawi. This was a planned analysis using data from a previously published prospective observational study that compared quality of life (QOL) and function for adults with closed femoral shaft fractures treated with IMN or ST in Malawi (Chokotho et al. 2020). Adult patients were recruited from 6 hospitals in Malawi: Queen Elizabeth Central Hospital (QECH), Kamuzu Central Hospital, Beit Cure International Hospital, and Chiradzulu, Thyolo, and Chikwawa district hospitals. Patients were excluded using the following criteria: (1) age less than 18 (n = 4), (2) polytrauma or multiple injuries, defined as any additional injury requiring admission on its own merits (admission was used as a proxy for severity of injury since it was not possible to calculate injury severity scores; n = 7), (3) pathological fractures (n = 2), (4) open fractures (n = 5), (5) clinical evidence of infection at the surgical site before or during surgery (n = 1), and (6) prior surgery involving the affected femur (n = 1).

Study participants were treated using ST or IMN at the discretion of the treating surgeon or orthopedic clinical officer (OCO). OCOs are non-physician clinicians trained to provide nonoperative care for orthopedic conditions and emergency orthopedic surgery for selected cases, such as acute infections and open fractures (Mkandawire et al. 2008). Follow-up assessments for both groups were performed at 6 weeks, 3 months, 6 months, and 1 year after the injury. At each follow-up, the patients were assessed clinically, the EQ-5D-3L was administered, and radiographs taken when feasible.

### Treatment technique

The SIGN nail (Zirkle and Shahab 2016) was used in all IMN patients. This is a solid locking IM nail that can be inserted without the need for a fracture table or intraoperative fluoroscopy. The SIGN nail was inserted antegrade using open reduction, with the patient in the lateral position on a standard operating table.

All ST patients had straight leg extension skeletal traction with a Steinmann pin inserted into the proximal tibia under local anesthesia, using an aseptic technique and a stirrup to connect the rope that was used to position the weights. The weights were positioned either using a bar or pulleys, or by placing the rope directly over the end of the bed, depending on the type of bed and equipment available at the hospital.

### Effectiveness data

We measured the effectiveness of each treatment strategy using quality-adjusted life years (QALYs) based on the EQ-5D-3L (Rabin and Charro 2001). At each follow-up time point, research assistants administered the EQ-5D-3L questionnaire to the study participants. The EQ-5D-3L is a tool used to measure health-related quality of life (HRQoL) that has been translated to Chichewa and validated for use in Malawian orthopedic patients (Chokotho et al. 2017). Utility scores were calculated using EQ-5D-3L responses based on data from the Zimbabwean population value set (Jelsma et al. 2003). QALYs were calculated from the utility scores using the area under the curve (AUC) method (Billingham et al. 1999). The AUC was calculated by multiplying the EQ-5D score at each time point by the midpoint duration between follow-up visits to capture both pre- and post-visit health states. We calculated QALYs for each of the 4 health states (successful IMN, unsuccessful IMN, successful skeletal traction, and unsuccessful skeletal traction). There was no measurable difference between groups in EQ-5D-3L index score at 1 year after treatment, hence a 1-year time horizon was used.

### Costing data

Direct medical and overhead costs and indirect patient costs were estimated. Direct costs were estimated using time-and-motion analysis and included procedure personnel and supplies; ward personnel; medications and investigations; surgical implants; and instruments. Even though the SIGN nail is distributed free of charge in LMICs, to be more conservative in our economic analysis we included the hardware production cost, which was obtained directly from the manufacturer. The figure includes both manufacturing and distribution costs. Overhead costs included food, building maintenance, renovation, cleaning and sanitation, bedding, stationery, uniforms, protective wear, and staff training. The direct medical and overhead cost data was collected on a subset of patients in the main clinical study at 1 of the 6 sites (QECH) (Diab et al. 2019). Hourly salaries for personnel were calculated by dividing mean annual salary by the product of 9-hour days, which is the average working hours for public hospitals in Malawi, and 251 working days per year. Further details on how the direct costs were calculated have been published earlier (Diab et al. 2019). All costs were presented in 2017 US$. Outpatient costs included clinic personnel, physiotherapy, and radiography costs. Indirect costs included patient lost productivity, and patient transportation, meals, and childcare costs associated with hospital stay and follow-up visits. We calculated costs associated with lost productivity for patients who reported either formal or informal employment prior to injury. Employment was scored as a binary value at each follow-up time point. Using midpoints between follow-up visits before and after the follow-up time point, overall lost productivity was weighted by the sum of weeks of reported unemployment, with a maximum of 52 weeks. The costs associated with productivity loss were calculated using a standardized wage for Malawi, adjusted using purchasing power parity to US$(World Bank 2019b). Patients were interviewed to estimate transportation, meal, and childcare costs. Resource utilization for each treatment group is given in Table 1.

**Table 1.** Resource utilization for each treatment group including both direct and indirect costs excluding the cost of failed traction or surgery requiring reoperation expressed as US$

Factor	IMN	ST
Direct costs		
Inpatient		
Ward personnel	264	445
Overhead	116	196
Surgical implants	136	0.0
Investigations	38	288
Procedure personnel	24	2.7
Procedure supplies	8.5	3.6
Instruments	8.7	0.2
Medications	2.2	1.6
Total	597	678
Outpatient		
Clinic personnel	1.0	0.8
Physiotherapy personnel	2.6	1.2
Radiography	41	29
Total	45	31
Total direct costs	642	709
Indirect costs		
Lost productivity	454	610
Transportation	4.0	4.0
Meals	3.4	0.7
Childcare costs	20	25
Total indirect costs	480	640
Total costs (direct + indirect costs)	1,122	1,349

### Decision-tree model

**Figure 1.**
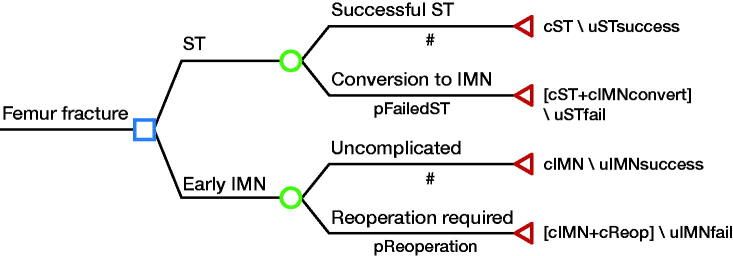
Decision-tree model of possible outcomes after ST and IMN treatment of femoral shaft fractures. Costs and effectiveness of each pathway are presented at the end of each potential pathway.

We constructed a simple decision-tree model (Figure 1) to compare the 2 treatments using TreeAge Pro 2020 (Pro 2019). In the ST treatment strategy, there were 2 potential outcomes: (1) successful traction, or (2) failure of treatment with conversion to IMN. Successful traction was defined as complete fracture union after treatment with ST. Failure of ST treatment was defined as either delayed union or non-union of the fracture requiring conversion to IMN. Patients treated in the IMN group had 2 potential outcomes: (1) successful IMN, or (2) failure of treatment with reoperation. The diagnosis of delayed union was made by the treating clinician if, at 6 weeks or more post-injury, there was still tenderness and mobility at the fracture site and no ­radiological evidence of callus formation. Non-union was defined as no evidence of fracture healing both clinically and radiologically after at least 3 months on ST or 6 months after IMN.

The primary outcome of the analysis was the incremental cost-effectiveness ratio (ICER), which was calculated by dividing the difference in cost by the difference in utility between the 2 treatment groups. The inputs for the model are given in Table 2. There was no difference in EQ-5D-3L index scores at 1 year in the primary study, hence a 1-year time horizon was used. Although typically 3% discounting would be applied, because of the 1-year time horizon we did not apply discounting. Both payer and societal perspectives were considered in the base case.

**Table 2.** Inputs for the decision-tree model

Factor	Mean (95% CI)	SE	Distribution type	Ref. ^a^
Costs				
IMN				
Direct inpatient cost	597 (543–651)	27	Gamma	D
Direct outpatient cost	45–	–	–	C
Cost of reoperation	900 (600–1,200)	–	Uniform	^b^
Indirect cost (societal)	480–	–	–	C
ST				
Direct inpatient cost	678 (624–732)	28	Gamma	D
Direct outpatient cost	31–	–	–	C
Cost of IMN after failed ST	649 (563–735)	49	Gamma	D
Indirect cost (societal)	640–	–	–	C
Utilities				
IMN				
Utility successful IMN	0.77 (0.72–0.83)	0.03	Beta	C
Utility after reoperation	0.71 (0.61–0.81)	0.05	Beta	E
ST				
Utility successful ST	0.72 (0.66–0.79)	0.03	Beta	C
Utility failed ST	0.69 (0.61–0.76)	0.04	Beta	C
Probabilities (%)				
IMN				
Probability reoperation	1.8 (0.0–5.3)	0.02	Beta	C
ST				
Probability failed ST	30 (22–38)	0.04	Beta	C

**^a^**C – Chokotho et al. 2020; D – Diab et al. 2019; E – Eliezer et al. 2017

**^b^** Assumed reoperation cost 1- to 2-fold higher than index surgery.

### Sensitivity analysis

We made both deterministic and probabilistic sensitivity analyses to assess which parameters are most important for the ICER and the uncertainty of the ICER input parameters. A tornado diagram was used to perform multiple 1-way sensitivity analyses assessing the relative influence of each model input on the ICER across a range of plausible input values based on the upper and lower limits of 95% confidence intervals (CI). 1-way sensitivity analyses were presented independently where appropriate. A multivariate probabilistic sensitivity analysis (PSA) was completed by performing 10,000 iterations of the model with a unique value for each input drawn from a probability distribution. The distributions used and standard errors are shown in Table 2. In general, costs were represented using a gamma distribution (range 0 to ∞) while probabilities and utilities were represented with a beta distribution (range 0 to 1). The results of the PSA are presented as an ICER scatter plot, which visually demonstrates the outcome of each iteration of the PSA as a point on the cost-effectiveness plane.

### Ethics, funding, and potential conflicts of interest

The study was approved by the College of Medicine Research Ethics Committee in Malawi, and the Western Norway Regional Research Committee and University of California San Francisco Institutional Review Boards. Written informed consent was obtained from all patients in the study. The study was funded by the James O. Johnston Research Grant, a PhD grant by Norad through the Norhed Project, and the Institute of Global Orthopedics and Traumatology (IGOT), University of California San Francisco. Author DS is a non-paid member of the Board of Directors for SIGN Fracture Care International. The rest of the authors declare no conflicts of interest.

## Results

We used data from 187 patients who completed 1-year follow-up to estimate utilities and probabilities, including 55 cases treated with a SIGN intramedullary nail (IMN) and 132 cases treated with ST. The overall total QALYs at 1 year were higher after IMN compared with ST (Table 3).

**Table 3.** Output from decision-tree model including incremental cost (US$) and effectiveness with 95% confidence intervals

Skeletal traction	
Utility	0.71 (0.66–0.76)
Payer cost	903 (828–986)
Societal cost	1,543 (1,469–1,625)
Early IMN	
Utility	0.77 (0.71–0.82)
Payer cost	659 (599–729)
Societal cost	1,139 (1,080–1,210)
Incremental utility	0.06
Incremental payer cost	–244
Incremental societal cost	–404

We used data on a subset of 65 patients treated at QECH (38 IMN, 27 ST) to estimate direct costs. The total direct cost of treatment was higher in the ST group compared with IMN (see Table 3). The total societal cost was higher for ST ($1,543; CI $1,149–$1,625) than IMN (1,139; CI $1,080–$1,210). Based on higher costs from both payer and societal perspectives, and lower utility with ST, IMN was the dominant strategy.

### Sensitivity analysis

**Figure 2.**
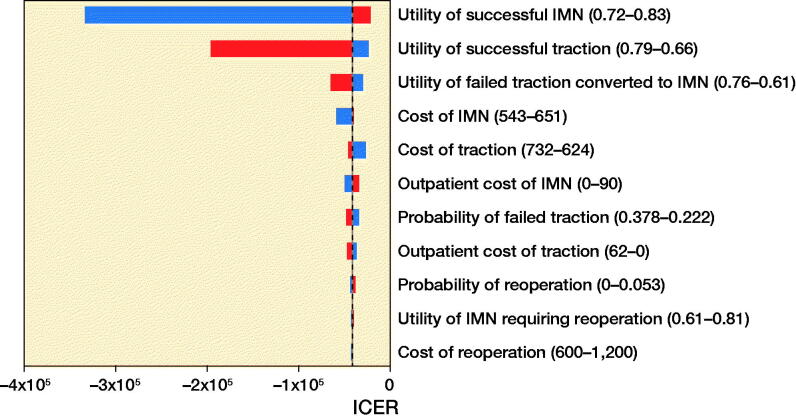
Tornado diagram demonstrating influence of each variable on the ICER across a plausible range of inputs based on the upper and lower bound of 95% confidence interval. Dotted line represents ICER per QALY gained for the base case.

The Tornado diagram (Figure 2) shows that the ICER was most sensitive to effectiveness of successful IMN, followed by effectiveness of successful traction. No change in the range of values for any of the variables resulted in IMN being less cost-effective than ST.

**Figure 3.**
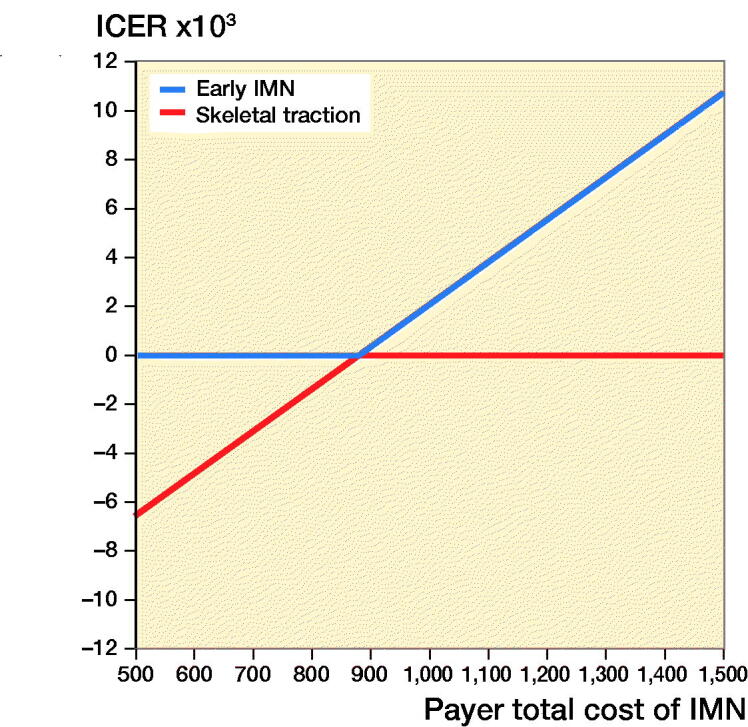
1-way sensitivity analysis on the payer total cost of early intramedullary nailing and ICER.

**Figure 4.**
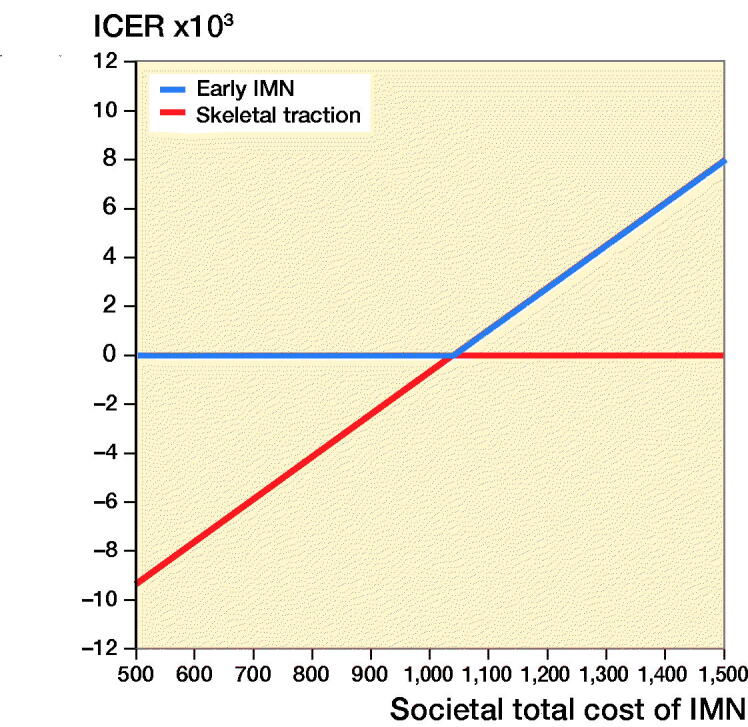
1-way sensitivity analysis on the societal total cost of early intramedullary nailing and ICER.

Figures 3 and 4 show the 1-way sensitivity analysis varying the total cost of IMN on ICER from the payer and societal perspectives respectively. IMN was dominant (more effective, less costly) up to a total procedure cost of $880 from the payer perspective or $1,035 from the societal perspective. Focusing specifically on the cost of the intramedullary implant, surgery was cost saving up to a nail cost of $472 from the payer perspective or $691 from a societal perspective.

### Probabilistic sensitivity analysis

**Figure 5.**
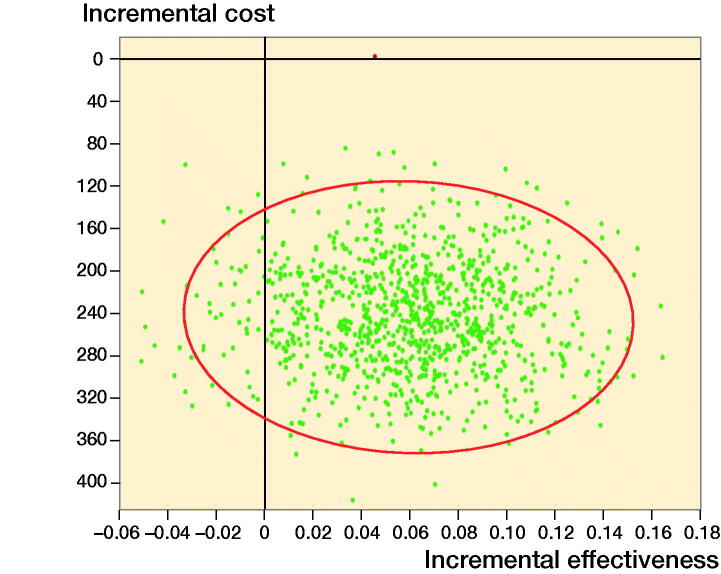
ICER scatter plot demonstrating output from the probabilistic sensitivity analysis.

The ICER scatter plot (Figure 5) shows that IMN was cost saving and more effective (dominant) in 93.8% of simulations.

## Discussion

This study found that treatment of adult femoral shaft fractures with IMN was more cost-effective than with ST in Malawi. Sensitivity analyses showed more than 90% certainty that this conclusion is true, and will remain true for IMN procedural costs of less than $880 and $1,035 from the payer and societal perspectives, respectively. Although there were no substantial differences in effectiveness between treatment modalities at 1 year, there were small differences at the other time intervals (Chokotho et al. 2020). The cost of IMN was lower and utility higher compared with ST; IMN is therefore the dominant approach from both societal and payer perspectives.

The finding of lower cost of IMN compared with ST has been reported by previous studies. Gosselin et al. (2009) found lower costs for IMN compared with ST, even after accounting for re-nailing costs following infection or non-union. Gosselin also reported better union rates with IMN than ST. Similarly, both Opondo et al. (2013) and Kamau et al. (2014) found IMN to be less costly with better healing and functional outcomes than ST among patients with femoral shaft fractures in Kenya. However, the time horizon in these studies ranged from 12 to 16 weeks, limiting the assessment of non-union. Further, there was no measure of patient-reported outcomes or preference-weighted instruments, such as the EQ-5D, and cost was measured only from the payer perspective. While our study found similar EQ-5D scores between the 2 groups at 1-year follow-up, a high percentage (30%) of the patients treated with ST required conversion to IM nailing due to either delayed union or non-union (Chokotho et al. 2020). Had these patients not converted to IM nailing, it is likely that they would have had substantially worse EQ-5D scores at 1-year, meaning this conversion likely mitigated some of the negative effects as patients were “rescued” from skeletal traction complications by conversion to IMN. In this sense, the conversion biased the effectiveness estimate towards the null hypothesis.

Patients treated with ST in Malawi are normally admitted to hospital for at least 6 weeks whereas those treated with IMN have an average length of stay of 17 days (Diab et al. 2019). In Malawi, patients do not pay service fees in public hospitals, therefore prolonged hospital stay is likely to have cost implications from the governmental payer perspective. A treatment method like IMN, which is both cost-saving and more effective, is certainly worth prioritizing to optimize the limited health budget.

Prolonged hospital stay is also likely to have financial implications for the patients and their guardians, who usually accompany patients in the hospital during the entire admission period. Due to the lack of nursing staff in Malawi, it is customary for these guardians, who are typically family members, to serve as the primary caregiver for patients during their hospitalization, with both patients and caregivers incurring substantial indirect costs of lost productivity, and hospital-related expenses. This is the first study that has evaluated the cost-effectiveness of femoral shaft fracture treatment with IMN and ST from the societal perspective. Haug et al. (2017) found that patients treated with skeletal traction complained that prolonged hospitalization caused severe financial strain because patients and their families were unable to engage in income-generating activities. In addition, they found that there was increased out-of-pocket expenditure while in hospital. Survival mechanisms to keep up with the increased expenditure included selling their property and borrowing money, sometimes with high interest rates (Damme et al. 2004, Kohler et al. 2017). Therefore, if hospital stays can be reduced through IMN, this treatment has cost-saving potential from both the governmental payer and the societal perspective.

SIGN Fracture Care International currently donates intramedullary nails free of charge to many hospitals in LMICs, including Malawi. This fact increases the potential cost saving beyond this study’s estimates, since our analysis included the cost of the IM nail. Cost-effective interventions are, however, not always affordable and accessible, and such is the case for Malawi where provision of operative fracture treatment is not universal in public hospitals. Future studies should include budget impact analyses assessing the affordability of adopting a new intervention from the payer’s perspective (Sullivan et al. 2014), thereby evaluating the opportunity costs and relevant benefits associated with choosing IM nailing as first-line treatment over ST.

Our study had several limitations. First, as it was not a randomized study, there were likely unmeasured confounding variables. However, the body of evidence supporting IM nailing (Amihood 1973, Gosselin et al. 2009, Opondo et al. 2013, Kamau et al. 2014), would likely make an RCT unethical to perform. Second, loss to follow-up at the different time points could lead to selection bias, thereby affecting our findings. However, there was no differential loss to follow-up as the proportions in both groups were similar. The results in our model were validated by univariate sensitivity analysis and probabilistic sensitivity analysis and both analyses showed that IMN was the cost-effective treatment approach. Third, the time horizon of 1 year used in this study may have been too short. As such we may have missed long-term QALY gains. However, effectiveness was similar between the 2 treatment groups at 1 year, likely because those who failed ST treatment, and were likely to have a poor outcome if left untreated, were switched to treatment with IMN. Conversely, in a setting where IMN is not offered, ST is likely to result in substantial loss of QALYs. Another limitation is that the decision tree in our analysis did not include all possible pathways or complications that represent the course of outcomes after treatment. Only delayed union and non-union were considered because cost data for other complications was not available. The majority of patients in this study were recruited from government-run hospitals, and so the findings may not be applicable to patients treated in private care facilities. However, ST treatment is not routinely offered in private hospitals, where all patients are treated with IMN, and the majority of the population in Malawi does not have medical insurance and therefore uses public hospitals where services are free at point of care. Thus, our findings are applicable to the majority of health facilities in the country.

In conclusion, despite its limitations, our study has shown that IMN is more effective and costs less than ST, and therefore scale-up of IMN may be an efficient use of limited healthcare resources in low-income countries. Our findings are relevant to healthcare policymakers and other stakeholders to justify and advocate for improved surgical capacity so that patients with femoral shaft fractures are treated with intramedullary nailing rather than skeletal traction.
